# Time capsules in natural sediment archives—Tracking phytoplankton population genetic diversity and adaptation over multidecadal timescales in the face of environmental change

**DOI:** 10.1111/eva.12513

**Published:** 2017-09-09

**Authors:** Marianne Ellegaard, Anna Godhe, Sofia Ribeiro

**Affiliations:** ^1^ Department of Plant and Environmental Sciences University of Copenhagen Frederiksberg Denmark; ^2^ Department of Marine Sciences University of Gothenburg Gothenburg Sweden; ^3^ Glaciology and Climate Department Geological Survey of Denmark and Greenland (GEUS) Copenhagen K Denmark

**Keywords:** diatom, dinoflagellate, environmental change, marine, population genetics, resting stage, sediment record

## Abstract

Undisturbed records of resting stages produced in the past and stored in coastal sediments are very valuable to science, because they may provide unique insights into past evolutionary and ecological trajectories. Within marine phytoplankton, multidecadal time series of monoclonal strains germinated from resting stages have been established for diatoms (*Skeletonema marinoi*) and dinoflagellates (*Pentapharsodinium dalei*), spanning ca. a century. Phenotypic and genotypic analyses of these time series have revealed effects of past environmental changes on population genetic structure. Future perspectives include direct comparisons of phenotypes and genotypic data of populations, for example, by genomewide assays that can correlate phenotypic trends with genotypes and allele frequencies in temporally separated strains. Besides their usefulness as historical records, “seed” banks of phytoplankton resting stages also have the potential to provide an inoculum that influences present populations through “dispersal from the past” (the storage effect) and are important for adaptation to future environments through their standing genetic diversity.

## 1 INTRODUCTION

**Figure 1:**
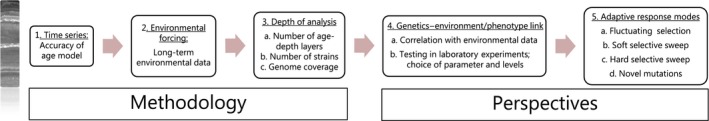
Overview over prerequisites for, and potential perspectives from, temporal records of phytoplankton to test adaption to environmental change. 1: The first requirement is a reliable age model. This can be achieved by radio‐isotope dating or, in some cases, by datable layers or other remains. To the left is an X‐ray image showing layers in a sediment core. 2: If a study is aimed at testing the effect of environmental forcing, temporal data on the selected environmental parameter(s) are needed, such as monitoring time series or proxy data (see text). 3: It can be difficult, but is important, to establish large numbers of strains from each age layer. So far, such studies on temporal genetic structure have been based on microsatellite data, but in the near future we will likely have data from additional markers, that span the entire genome, for example, SNPs from resequenced genomes, which can be used to trace adaptive trends as well as neutral processes. 4: Linking temporal genetic structure with environmental factors and/or phenotypic adaptation by (a) temporal correlation with environmental data; (b) testing age series of strains at a range of parameters(s) in controlled experiments. 5: Examples of possible genetic responses to temporal environmental change that can be derived from genomic data obtained from consecutive time periods

The plankton comprises organisms that are free‐floating in the water column and have limited capability for independent movement. A main functional group in the plankton is the phytoplankton, that is, the photosynthesizing fraction, which broadly speaking comprises the planktic microalgae. Many planktic organisms form a resting stage as part of their life cycle and these resting stages sink through the water column and may accumulate in the sediments (e.g., Ellegaard & Ribeiro, 2017). Such stages may be buried in the sediment and under the right conditions they will over time constitute a time series of individual cells. Hence, going down into the sediment archive will be equivalent to going back in time (Figure 1; Ellegaard et al., 2013). To achieve this, the sediment must be (mostly) undisturbed after deposition, by, for example, weather, human activity, strong currents or bioturbation by benthic animals. Thus, areas of net‐sediment deposition and low oxygen levels in the sediment will be most suited for the establishment of such time series of resting stages (Ellegaard et al., 2013).

Undisturbed sediment records are temporal archives of a multitude of different organisms and they have been utilized extensively in paleoecology, which is the hind‐cast of environmental conditions based on temporal series of traces of biological organisms. However, some plankton resting stages have been shown to remain intact and viable in the archives over time scales of years to decades (e.g., Ellegaard et al., 2013; examples in Table 1). The temporal records of these constitute a time series preserving entire cells and thus present opportunities for studying both genomic and phenotypical characteristics of populations through time. Studies on longevity in the sediment record have documented maximal duration of viability for a range of organisms (see Table 1 for an overview of ranges for diatom and dinoflagellate species). In recent years, this has been extended to include the establishment of clonal strains from germinated resting stages of different age layers and subsequently studying genetic and phenotypic aspects of strains or populations representative of the time series. This approach with associated methodology is in this special issue referred to as *resurrection ecology*.

**Table 1:** Overview of records of maximal in situ duration of viability of diatoms and dinoflagellates

Species	Environment	Sediment core depth (cm)	Estimated age (years)	Reference
Diatoms				
*Melosira (Aulacoseira) italica* (Ehrenberg) Simonsen	Lake	25–35	175–275^a^	Stockner and Lund (1970)
*Skeletonema marinoi* Sarno & Zingone et al.	Coastal marine (fjord)	44	>55	McQuoid et al. (2002)
		22	>80	Härnström et al. (2011)
*Chaetoceros socialis* H. S. Lauder		44	>55	McQuoid et al. (2002)
*Detonula confervacea* (Cleve) Gran				
*Chaetoceros* Ehrenberg spp.		39	80	Ellegaard et al. (2013)
Dinoflagellates				
*Alexandrium* Halim spp.	Coastal marine (bay)	63	61	Feifel, Fletcher, Watson, Moore, and Lessard (2015)
*Alexandrium tamarense* (Lebour) Balech		32	ca. 100	Miyazono, Nagia, Kudo, and Tanizawa (2012)
*Scrippsiella* Balech ex A.R.Loeblich III spp.		63	61	Feifel et al. (2015)
*Pentapharsodinium dalei* Indelicato & Loeblich III	Coastal marine (fjord)	44	>55	McQuoid et al. (2002)
		37	ca. 90	Lundholm et al. (2011)
*Lingulodinium polyedrum* (F. Stein) J. D. Dodge		37	ca. 90	Lundholm et al. (2011)
*Spiniferites* G.A. Mantell spp.		17	30	Ellegaard et al. (2013)

a Age estimated by estimating sedimentation rate.

In this synthesis, we review the research on genetic population diversity and structure of phytoplankton over multidecadal time scales and summarize the results of these studies. We explore what impact past environmental changes may have had on either genetic structure and/or phenotypical characteristics of these organisms and suggest future perspectives for this emerging field.

## 2 REVIEW

### 2.1 Temporal records of population genetics and physiology of diatoms and dinoflagellates

So far, the studies published on temporal series of phytoplankton strains have focused on diatoms and dinoflagellates, two important groups of phytoplankton. This may be partly due to the fact that these two groups have robust resting stages allowing many of their species to remain viable over multiple decades. However, other phytoplankton also exhibit this trait (see, e.g., Ellegaard & Ribeiro, 2017; Ellegaard et al., 2013), but to our knowledge such temporal series of strains have not been established for other groups. The maximum age of viable cells reported for these two groups from isotope‐dated sediment cores is ca. 100 years (Table 1); however, data on temporal genetics and physiology at population level are available so far only for the diatom *Skeletonema marinoi* and the dinoflagellate *Pentapharsodinium dalei*.

These two species have been studied for various reasons: (i) they were found to have long temporal extent of viability in sediment cores and were found alive furthest down‐core in highest numbers in studies targeting all potentially viable dinoflagellate cysts (Lundholm et al., 2011) and diatoms (McQuoid, Godhe, & Nordberg, 2002) from cold‐temperate fjord sediments; (ii) the two species are easy to keep in culture; (iii) both species have a relatively wide geographical distribution (Kooistra et al., 2008; Rochon, de Vernal, Turon, Matthiessen, & Head, 1999) and (iv) these species are ecologically important in their native areas. This has particularly been demonstrated for *S. marinoi*, which is an important component of the spring bloom of diatoms in temperate areas (e.g., Godhe et al., 2016). Finally, considerations of availability, or development, of relevant population genetic markers are central. Although the number of population genetic studies of phytoplankton has grown dramatically in the last decade (see, e.g., Lebret, Kritzberg, Figueroa, & Rengefors, 2012; Tesson, Montresor, Procaccini, & Kooistra, 2014), species‐specific high‐resolution genetic markers have nonetheless been developed for relatively few phytoplankton species. For both *P. dalei* and *S. marinoi*, microsatellite genetic markers were developed primarily for studying temporal population genetic changes (Almany et al., 2009; Lundholm, Nielsen, Ribeiro, & Ellegaard, 2014).

Clonal strains from past populations of these two species have been successfully established in the laboratory by germinating resting stages from undisturbed sediments deposited up to 100 years ago (Härnström, Ellegaard, Andersen, & Godhe, 2011; Lundholm, Ribeiro, Godhe, Nielsen, & Ellegaard, 2017) in coastal areas, which have experienced marked shifts in environmental conditions during the past century. Therefore, experiments on the revived strain series have been done to test hypotheses of the effects of these environmental changes on ecophysiological preference of the strains (Ribeiro, Berge, Lundholm, & Ellegaard, 2013). Also, changes in population genetic structure have been linked to past environmental shifts (Härnström et al., 2011; Lundholm et al., 2017).

One study (Härnström et al., 2011) tested population genetic structure of *Skeletonema marinoi* from five age‐depth layers in the hypereutrophic Danish inlet Mariager fjord spanning >80 years during which very large changes in nutrient loading have occurred (Ellegaard et al., 2006). The population genetic analysis was based on microsatellite markers (Almany et al., 2009; Godhe & Härnström, 2010). Overall, the data indicated large genetic diversity in all layers and a more or less stable structure, but one layer (from ca. 1980) displayed significant differentiation and reduced diversity. This layer corresponded to the timing of an extreme anoxic event in Mariager fjord. The main difference in population genetic structure, however, was between the strains inside Mariager fjord, which is enclosed, and heavily eutrophied, and strains established from sediment from the Kattegat, the open waters outside the fjord. The conclusion based on this study, and later supported by a common garden experiment (testing the effect of environment by moving each of the populations from their native environments into a foreign environment; Sildever, Sefbom, Lips, & Godhe, 2016), is that the *S. marinoi* population inside the fjord is specifically adapted to the local conditions and that, although there is water exchange with the Kattegat, *S. marinoi* from outside the fjord did not mix with the inner fjord population to any significant degree through the entire time period.

A second set of studies focused on the dinoflagellate *Pentapharsodinium dalei* germinated from dated sediment core layers from Koljö Fjord, a cold‐temperate fjord on the Swedish coast. This fjord is less affected by anthropogenic eutrophication than Mariager fjord, but paleoecological studies have documented environmental shifts in the fjord coinciding with atmospheric variability linked to the North Atlantic Oscillation (NAO), which also affects the abundance of *P. dalei* cysts in the sediments (Harland, Nordberg, & Filipsson, 2004). Two studies explored population genetic structure and ecophysiology of *P. dalei* over ca. 100 years. Ribeiro et al. (2013) tested the growth performance of *P. dalei* at two different salinity levels (15 and 30), corresponding to the maximal and minimal salinities expected in negative and positive modes of the NAO, respectively. The study showed that there was large variability between strains but that there were no significant differences in response to salinity or in maximal pH tolerance between the three tested layers (strains from all layers grew best in the high salinity treatment). Lundholm et al. (2017) analyzed population genetic structure using six microsatellites markers (Lundholm et al., 2014). Similar to the study on *S. marinoi* in Mariager fjord, the data showed large genetic diversity in all layers and an overall stable population structure. However, two subpopulations shifted in dominance during the ca. 100 year time span, with one subpopulation becoming dominant during conditions of predominantly negative NAO.

### 2.2 Prerequisites and methodologies

The specific methodologies used are of great importance for the scope and interpretations of phytoplankton genetic diversity and adaptation over multidecadal timescales (Figure 1). An essential requirement is that the sediment cores, from which the isolated strains derive, are taken from undisturbed, net‐depositional environments and that it is possible to obtain a reliable chronology for the sediment records.

The most robust methods for dating sediment records involve radioactive isotopes. At the time scales relevant here, the most commonly used method is dating by ^210^Pb, often supported by ^137^Cs (e.g., in Northern Europe), which, under ideal circumstances, can be applied to sediments up to ca. 120–150 years old (Andersen, 2017). Older (over 500 years) sediments are often dated using ^14^C. There are a number of conditions that need to be met for a reliable age‐depth model to be achieved. In coastal settings, the two main requirements are that the sediment must be fine‐grained and bioturbation must be absent or very limited (Andersen, 2017). These two requirements are often met in the same types of depositional environments that are best suited for concentration and preservation of living sediment stages of phytoplankton, for example, areas with net deposition (low physical forcing) and anoxia or low oxygen (which both limits bioturbation and tends to preserve viability; Ellegaard et al., 2013). Good age‐depth models with low uncertainties are the basis for the precision and robustness of the environmental interpretations (Figure 1.1).

Establishing monoclonal strains of algae from sediment cores is labor intensive. After the challenges in obtaining sediment cores with robust chronologies, the cores must be subsampled, taking particular care to minimize the risk of contamination or smear between layers. This can be accomplished by discarding the outer mm of each slice, or subsampling from the center of each slice. Then, cells must be individually isolated from either rinsed sediment samples (e.g., dinoflagellate cysts) or enriched sediment slurries (e.g., diatoms). In the case of sexual stages such as dinoflagellate cysts, this isolation must be done twice to achieve monoclonal strains (first by isolating the diploid resting cyst and thereafter isolating a single haploid vegetative cell). Both the work involved and the availability of viable cells back in time limit the number of strains that can be resurrected (Figure 1.3). However, the immense genetic and phenotypic diversity within phytoplankton populations makes it very important to put effort into resurrecting as many different clones as possible. Microsatellite‐based data from 147 monoclonal strains of *P. dalei* and 158 monoclonal isolates of *S. marinoi* revealed that 98%–100% of the genotypes revived from sediment archives were unique (Härnström et al., 2011; Lundholm et al., 2017). In addition, as in all cultivation‐based work, there is a risk of biased representation as all viable cysts might not germinate at the chosen conditions and further, some pheno‐ and/or genotypes may be eliminated over time.

The studies published so far have used microsatellite markers to study population genetic structure. Microsatellites are very suitable for such studies, but due to difficulties and cost of development, so far <10 different loci have been used, making the coverage of the genome limited and restricting the data to noncoding sites. Ongoing studies of *P. dalei* from a Greenlandic fjord (S. Ribeiro, S. Hardardottir, M. Ellegaard, T.J. Andersen & K. Rengefors, in prep.) and *S. marinoi* (Godhe, unpublished) from the Swedish inlet Havstens fjord and Mariager fjord attempt to increase the analysis depth by employing genomewide methodologies. Thus, in the study on *S. marinoi* the entire genomes of hundreds of individuals have been resequenced and single nucleotide polymorphisms (SNPs) have been identified in candidate genes. In the study on *P. dalei*, amplified fragment length polymorphisms have been developed for studying temporal population genetic structure (Figure 1.3).

The availability of temporal data series on environmental conditions in a potential sampling site is another central factor (Figure 1.2). To test for adaptation in the face of environmental change, the timing, level and extent of environmental change must be known. In a few cases, multidecadal time series of environmental monitoring data are available (e.g., in Mariager Fjord, Fallesen, Andersen, & Larsen, 2000), in other cases, and further back in time, reconstructions of environmental conditions based on paleoenvironmental data (e.g., Harland et al., 2004 for Koljö Fjord; Ellegaard et al., 2006 for Mariager Fjord), or on land‐use changes, may be available (e.g., Andersen et al., 1998; Ellegaard et al., 2006). Ideally, detailed environmental data should be available for the study site (local‐scale variability), although regional trends can also be useful to identify possible drivers of population changes. When working with sediment cores, there is a broad range of proxies that can be analyzed in parallel to single‐cell isolation (e.g., microfossil assemblages and biogeochemical tracers), which will allow for an environmental reconstruction based on the same sediment record.

## 3 DISCUSSION AND FUTURE PERSPECTIVES

### 3.1 Reconstructing phytoplankton adaptation to environmental change

Given the short mitotic generation time of phytoplankton and the large population sizes and genetic standing stock, phytoplankton resurrected from sediment cores provide a splendid material for studying adaptation to past and ongoing changes. Phytoplankton populations germinated from discrete layers of a sediment record can be kept side by side in the laboratory. This allows direct comparisons of phenotypes and genotypes of original and adapted populations. Establishing populations from several discrete layers permits trend analyses of phenotypic changes and changes in allele frequencies. For ecophysiological studies, it is crucial to select experimental treatments that mimic past or ongoing environmental change, and the phenotypic trait that best illustrates adaptive changes (Figure 1.4). When, for example, eutrophication is an important aspect, different nutrient concentrations or light intensities can be used. If thermal or CO_2_ adaptations are of interest, different temperatures or *p*CO_2_ can be tested (Lohbeck, Riebesell, & Reusch, 2012; Schlüter et al., 2014). One could either expose the populations to ecologically relevant maximum and minimum nutrient concentrations, irradiance, temperatures or *p*CO2 (as done with salinity in Ribeiro et al., 2011), or expose individuals to gradient of the manipulated parameters to assess the reaction norms of the populations. A significant shift in reaction norms between temporally separated populations is likely a strong indicator of an adapted population. The phenotypes most commonly used as response proxies among single celled protists are the extent of the lag phase, the growth rate and the growth efficiency (Blomberg, 2010). Several recorded phenotypic traits will more likely pick up a significant signal of adaptive change (Figure 1.5). Phenotypic novelties in the adapted populations are presumably a result of selection from the genetic standing stock (soft selective sweeps), or a result of mutations or new genotypes, which have entered the population through migration (hard selective sweeps) (Krehenwinkel, Rödder, & Tautz, 2015). By using either reduced representation libraries and high‐throughput sequencing for organisms with large genomes or resequencing for organisms with smaller genomes, SNPs can be detected and followed through time (i.e., down‐core). *F*_ST_ analyses will indicate outlier loci and may reveal genes that are affected by the environmental trend (Narum & Hess, 2011). Genomewide assays can correlate phenotypic trends with genotypes, and allele frequencies of selected SNPs may tell if the SNP is new to the population and has been introduced through migration or mutation or if it has gradually increased (or decreased) in frequency and thus is part of selection of the genetic standing stock (Figure 1.5).

A key potential of studies using temporal series of strains is to explore if and how populations can undergo evolutionarily significant changes in ecologically important traits on the same time scales as the changes that drive them (e.g., Becks, Ellner, Jones, & Hairston Jr., 2012), thus linking “ecological time” with “evolutionary time” via the study of eco‐evolutionary dynamics (e.g., De Meester & Pantel, 2014). Such studies have been performed in laboratory settings of, for example, prey–predator dynamics of *Chlamydomonas reinhardtii* P.A. Dangeard 1888 and a rotifer predator, which showed that changes in prey traits in response to predation influenced predator abundance over time scales of months and that there was a genetic component to these trait changes (Becks et al., 2012). So far, most laboratory experiments on eco‐evolutionary dynamics have used such simple systems, and there is a need for studies encompassing more of the complexity of natural systems. One potential avenue for this is employing the long‐term biological records of sediment archives. These have the potential to test for direction of selection (e.g., fluctuating selection; Figure 1.5.; Lundholm et al., 2017) and a future potential for linking changes in phenotype directly with environmental changes and further with changes in the genes coding for the phenotypic trait.

### 3.2 Resting stages in ecosystem functioning and resilience

As seen above, undisturbed records of resting stages produced in the past and stored in coastal sediments may provide unique insights into past evolutionary and ecological trajectories. Alongside with their potential as historical records, however, “seed banks” also have the potential to provide an inoculum that will influence present populations through “dispersal from the past.” In order for old resting stages buried in the sediment to germinate, the sediments have to be disturbed and resuspended, so that germination can be triggered. Hairston and Kearns (2002) argued that, although apparently contradictory, the historical and dynamic aspects of propagule banks could coexist in a same system, provided it contained net‐depositional (typically deeper sites) and erosional areas (near‐shore).

The maintenance of a phytoplankton “seed bank” that incorporates germinated cells from viable resting stages formed over several years implies that there is generation overlap. Overlapping generations in a fluctuating environment allow for the maintenance of diverse populations and the coexistence of competitors (the storage effect, Warner & Chesson, 1985). A viable seed bank may ensure the persistence of diverse populations even during years (potentially decades) of null recruitment, when conditions in the water column are adverse. This is of large significance for ecosystem resilience, and the potential for recovery from abrupt events such as environmental disasters (e.g., oil spills, acid rain; see also Ribeiro et al., 2011).

Climate change is an important driver of long‐term species evolution and extinction rates, and “seed banks” of microbial organisms have been suggested to play an important role in adaptation to future environments (Lennon & Jones, 2011). In the Baltic, ecophysiological experiments on genetically different dinoflagellate clones from distinct cyst banks showed that clones with higher growth rates at present conditions of temperature and salinity were not the same as those that perform better at conditions projected for the end of the century (Kremp et al., 2016). This further supports the idea that the standing genetic diversity in seed banks favors adaptation in the face of rapid environmental change, and indicates the potential of using the sediment record of viable cells to document responses to past variability and thereby predict the effect of future changes.
